# A retrospective study of the efficacy and safety of naldemedine for opioid‐induced constipation in thoracic cancer patients

**DOI:** 10.1111/1759-7714.14557

**Published:** 2022-07-05

**Authors:** Hisao Imai, Yukiyoshi Fujita, Eriko Hiruta, Takashi Masuno, Shigeki Yamazaki, Hajime Tanaka, Teruhiko Kamiya, Mitsuru Sandoh, Satoshi Takei, Kazuya Arai, Hiromi Nishiba, Junnosuke Mogi, Kyoichi Kaira, Koichi Minato

**Affiliations:** ^1^ Division of Respiratory Medicine Gunma Prefectural Cancer Center Ota Japan; ^2^ Department of Respiratory Medicine, Comprehensive Cancer Center, International Medical Center Saitama Medical University Hidaka Japan; ^3^ Division of Pharmacy, Gunma Prefectural Cancer Center Ota Japan; ^4^ Division of Pharmacy Fujioka General Hospital Fujioka Japan; ^5^ Division of Pharmacy Kiryu Kosei General Hospital Kiryu Japan; ^6^ Division of Pharmacy Haramachi Red Cross Hospital Higashiagatsuma‐machi, Agatsuma‐gun Japan; ^7^ Department of Pharmacy Tatebayashi Kosei General Hospital Tatebayashi Japan; ^8^ Division of Pharmacy Ota Memorial Hospital Ota Japan; ^9^ Division of Pharmacy Tone Central Hospital Numata Japan; ^10^ Division of Pharmacy Gunma Saiseikai Maebashi Hospital Maebashi Japan; ^11^ Division of Pharmacy Japan Community Health Care Organization (JCHO) Gunma Chuo Hospital Maebashi Japan; ^12^ Graduate School of Pharmaceutical Sciences Takasaki University of Health and Welfare Takasaki Japan; ^13^ Division of Pharmacy Hidaka Hospital Takasaki Japan

**Keywords:** clinical practice, efficacy, naldemedine, opioid‐induced constipation, peripherally‐acting mu‐opioid receptor antagonist

## Abstract

**Background:**

We conducted a multicenter, retrospective study on the efficacy and safety of naldemedine in thoracic cancer patients using opioids in clinical practice.

**Methods:**

We retrospectively evaluated thoracic cancer patients treated with naldemedine at 10 institutions in Japan. Clinical data of patients administered naldemedine between June 2017 and August 2019 were extracted from electronic medical records. Inclusion criteria were as follows: (i) patients hospitalized for at least seven days before and after naldemedine administration, and (ii) those whose frequency of defecation was entered in the medical records.

**Results:**

Forty patients were analyzed, and defecation frequency was observed for at least seven days before and after naldemedine administration. The response rate was 65.0% (95% CI: 50.2%–79.7%). The number of defecations increased significantly after naldemedine administration in the overall population, as well as among only those who defecated <3 times/week before naldemedine administration, and those that were administered ≥30 mg/day of morphine equivalent. Diarrhea was the most common adverse event in all grades, occurring in 11 patients (27.5%), of which 9 (81.8%) were grade 1 or 2. None of the patients experienced grade 4 or higher adverse events.

**Conclusion:**

The efficacy and safety of naldemedine for thoracic cancer patients in clinical practice were comparable with those of prospective studies, which suggest that naldemedine may be effective and feasible for most thoracic cancer patients.

## Graphical abstract

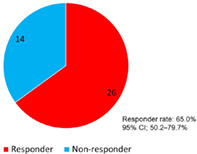

## INTRODUCTION

Thoracic malignancies include lung cancer, thymic epithelial tumors, and malignant pleural mesothelioma. Lung cancer accounts for a majority of thoracic cancers and is the most common cause of cancer‐related mortalities globally.^1^ As patients with lung cancer frequently experience significant pain, a better understanding of pain management strategies for this patient population is vital.^2^ Opioids are widely used as the standard treatment for moderate‐to‐severe cancer pain.^3^, ^4^ These drugs effectively treat cancer pain but are often limited by adverse effects that negatively impact the quality of life, sometimes leading to their discontinuation.^5^, ^6^ Opioid‐induced constipation (OIC) is one of the most frequent adverse events in patients using opioids and, without prophylaxis, occurs in more than 50% of patients using opioids.^7^, ^8^ It has been reported that most opioid analgesics can cause OIC, although the rate of occurrence varies according to the type of drug and route of administration.^9^, ^10^ Unlike other adverse events such as nausea and vomiting, continued administration of opioids has been reported not to increase the occurrence of OIC.^11^ Prolonged constipation increases the risk of abdominal pain, nausea, vomiting, anorexia, and delirium.^12^ These adverse events not only impair the quality of life but also create an obstacle in pain management by inhibiting the use of analgesic medications and preventing the use of rescue.^13^ Therefore, control of OIC is important for maintaining the quality of life of patients using opioids to treat cancer‐related pain. OIC, characterized as functional constipation, has been defined as a change from baseline bowel habits and defecation patterns following the initiation of opioid therapy.^14^ A recent study evaluated patterns of laxative prescription in patients with lung cancer using opioids. In the study, almost 90% of patients received inadequate or inappropriate OIC prophylaxis.^15^ Using the international Rome IV diagnostic criteria for OIC, a survey in Japan reported the incidence of OIC to be 56%.^16^ Another Japanese survey using the same criteria showed a 47.8% incidence of OIC in lung cancer patients.^17^

Opioids exert their analgesic effects primarily by activating opioid receptors in the central nervous system. The activation of μ‐opioid receptors in the intestinal tract suppresses normal bowel movement,^18^ and opioid‐induced intestinal dysfunction begins soon after opioid administration.^19^ Naldemedine is a peripherally acting μ‐opioid receptor antagonist that improves OIC by binding to opioid receptors in the gastrointestinal tract.^20^ This antagonist normalizes intestinal function by inhibiting the binding of opioids to the enteric nervous system without decreasing the analgesic effect of opioids. The efficacy and safety of naldemedine in cancer patients have been reported in randomized phase III trials, such as COMPOSE‐4 and COMPOSE‐5.^21^, ^22^ Major toxicities included diarrhea (19.6%), malaise (4.1%), vomiting (3.1%), and decreased appetite (3.1%), and 9.3% of the participants discontinued the drug due to adverse events.^21^

The incidence of OIC in patients with thoracic cancers, especially lung cancer, has been reported to be approximately 50%.^17^ Furthermore, OIC can occur quickly after opioid administration in patients with lung cancer, adversely impacting the quality of life.^17^ However, previous phase III trials included carefully selected participants (e.g., Eastern Cooperative Oncology Group performance status [ECOG‐PS] ≤ 2, and a cancer condition expected to remain stable for the extent of the study), and data on predictive factors or the detailed course of adverse events were lacking for patients with thoracic cancer. We previously conducted a survey on the use of naldemedine in clinical practice,^23^, ^24^ but there was insufficient evidence of its efficacy in thoracic cancer. Specifically, there are insufficient data from clinical practice involving patients with thoracic cancer who have poor performance status (PS) or those that are elderly. Therefore, we examined whether it is effective and safe to treat OIC in clinical practice in patients with thoracic cancer, including those with poor PS and those that are elderly. Thus, we conducted a multicenter, retrospective study on the efficacy and safety of naldemedine in patients with thoracic cancer using opioids in clinical practice.

## METHODS

### Patients

This multicenter, retrospective study of thoracic cancer patients treated with naldemedine was conducted among 10 institutions in Japan. The data of patients administered naldemedine between June 7, 2017, and August 31, 2019, were extracted from electronic medical records. Eligible patients were identified using electronic medical charts and pharmacy databases. Patients were included if they met the following criteria: (i) pathologically or cytologically diagnosed with a thoracic malignancy; (ii) naldemedine treatment initiated during hospitalization; (iii) naldemedine used in combination with opioids; and (iv) hospitalized for at least seven days before and after naldemedine administration. The frequency of defecation was noted in the medical records. Eighty‐three thoracic cancer patients who received naldemedine for the first time in conjunction with opioids during hospitalization were identified. Of those eligible patients, 43 patients who could not be observed for at least seven days before and after the start of naldemedine administration were excluded. Finally, 40 patients were included in the analysis (Figure S1). The data of 40 patients in current analysis are part of prevously described. We reviewed patient charts to collect data regarding baseline characteristics and responses to naldemedine. The study design was approved by the institutional review board of each participating institution, and the requirement for informed consent was waived owing to the retrospective nature of the study. However, the opportunity to refuse to participate through an opt‐out method was guaranteed.

### Treatment

The patients had not previously received naldemedine. In the current study, 0.2 mg of naldemedine was administered orally once a day with opioids. This treatment was continued until the occurrence of unacceptable toxicity, withdrawal of consent, or an attending physician judged termination to be necessary. The initiation and termination of naldemedine were decided by each attending physician.

### Assessment of treatment efficacy

We evaluated the number of defecations (times/week) seven days before and after naldemedine administration. A responder was defined as a patient with three or more defecations/week in the first seven days after naldemedine initiation and an increase of one or more defecations/week from the baseline. The baseline was the number of defecations during the week before naldemedine initiation. Adverse events were graded using the Common Terminology Criteria for Adverse Events version 5.0.

### Statistical analysis

Categorical variables were analyzed using Fisher's exact test. The Wilcoxon signed‐rank test was used to check normality and equal variances and test for correspondence between the two groups. Multivariate ordered logistic regression analysis was used to identify factors that predicted efficacy, and the results are expressed as odds ratios (ORs) and 95% confidence intervals (CIs). Differences were considered statistically significant at a two‐tailed *p*‐value ≤0.05. All analyses were conducted using JMP software for Windows, version 11.0 (SAS Institute).

## RESULTS

### Patient characteristics

Of the 40 patients included in the analysis, 36 died due to disease progression. The patient characteristics are shown in Table 1. The median age was 71 years (range, 38–88 years), with 12 (30.0%) patients aged ≥75 years. Additionally, 30 patients were male, and 10 were female. According to the Eastern Cooperative Oncology Group criteria, 10 patients (25.0%) had a PS of 0 or 1, 6 (15%) had a PS of 2, and 24 (60%) had a PS of 3 or 4, which was considered poor. In addition, 39 (97.5%) patients had lung cancer and one (2.5%) had malignant mesothelioma.

**TABLE 1:** Patient characteristics

Characteristic	*N* = 40
Sex	
Male/female	30 / 10
Median age at treatment (years) [range]	71 [38–88]
Performance status (PS)	
0 / 1 / 2/ 3 / 4	5 / 5 / 6 / 17 / 7
Primary tumor	
Lung cancer/malignant mesothelioma	39 / 1
Treatment before and during naldemedine administration^a^	
Anticancer agents^b^	11
Thoracic radiotherapy	9
Supportive care alone	20
Central nervous system metastasis	
Yes/No	10 / 30
Peritonitis	
Yes/No	1 / 39
Gastrointestinal obstruction	
Yes/No	0 / 40
History of abdominal surgery before starting naldemedine	
Yes/No	8 / 32
History of radiation to the abdomen and pelvic region before starting naldemedine	
Yes/No	7 / 33
Presence of diabetes mellitus	
Yes/No	6 / 34
Discontinuation of naldemedine within 7 days	
Yes/No	7 / 33
Use of laxatives before starting naldemedine administration	
Yes/No	36 / 4
Use of laxatives after starting naldemedine administration	
Yes/No	36 / 4
Regular use of antiemetic medication after initiation of naldemedine	
Yes/No or unknown	10 / 30
Abbreviated use of antiemetic agents after starting naldemedine	
Yes/No or unknown	8 / 32
Survival status at data cutoff date	
Death/Alive	36 / 4
Period to death from naldemedine initiation	
Median period (days) [range]	33.5 [9–578]

^a^ Within 3 weeks before starting naldemedine administration.

^b^ Detailed breakdown of anticancer agents therapy. The following treatment regimens were included: Carboplatin + paclitaxel 2, cisplatin + vinorelbin 1, cisplatin + pemetrexed 1, docetaxel + ramucirumab 1, gefibinib 1, alectinib 1, S‐1 1, pembolizumab 2, nivolumab

The use of opioids, laxatives, and antiemetic agents is shown in Table 2. The median regular opioid dose in oral morphine equivalents was 30 mg/day (range: 7.5–360 mg). Oxycodone was the most commonly used opioid in 22 patients (55.0%), followed by fentanyl in eight (20.0%). Moreover, 36 (90.0%) patients received concomitant laxatives, among whom 29 (80.6%) received magnesium oxide. Furthermore, 19 (47.5%) patients started naldemedine within 14 days of opioid initiation. Finally, 15 (37.5%) patients received concomitant antiemetic agents (regular or abbreviated use), among whom seven (46.6%) received metoclopramide.

**TABLE 2:** Administration of opioids, laxatives, and antiemetic agents

	N	(%)
Daily dose of opioids (mg)^a^		
<30	14	35.0
30–49	16	40.0
50–99	5	12.5
≥100	5	12.5
Regular use of opioids		
Oxycodone	22	55.0
Morphine	6	15.0
Fentanyl	8	20.0
Hydromorphone	4	10.0
Days from first opioid administration to initial naldemedine use (days)		
<4	2	5.0
4–7	1	2.5
8–14	16	40.0
15–28	7	17.5
29–99	8	20.0
≥100	6	15.0
Drugs of concomitant laxatives^b^		
Magnesium oxide	29	72.5
Sennoside	10	25.0
Bisacodyl	4	10.0
Lubiprostone	3	7.5
Sodium picosulfate hydrate	1	2.5
Sodium bicarbonate, sodium dihydrogen phosphate anhydrous suppository	6	15.0
Others	1	2.5
Drugs of concomitant antiemetic (regular and abbreviated) use^b^		
Metoclopramide	7	17.5
Domperidone	1	2.5
Prochlorperazine	5	12.5
Olanzapine	2	5.0
Others	3	7.5

^a^ Oral morphine equivalent to regular opioids.

^b^ Total number of patients.

### Treatment efficacy and safety

**FIGURE 1:**
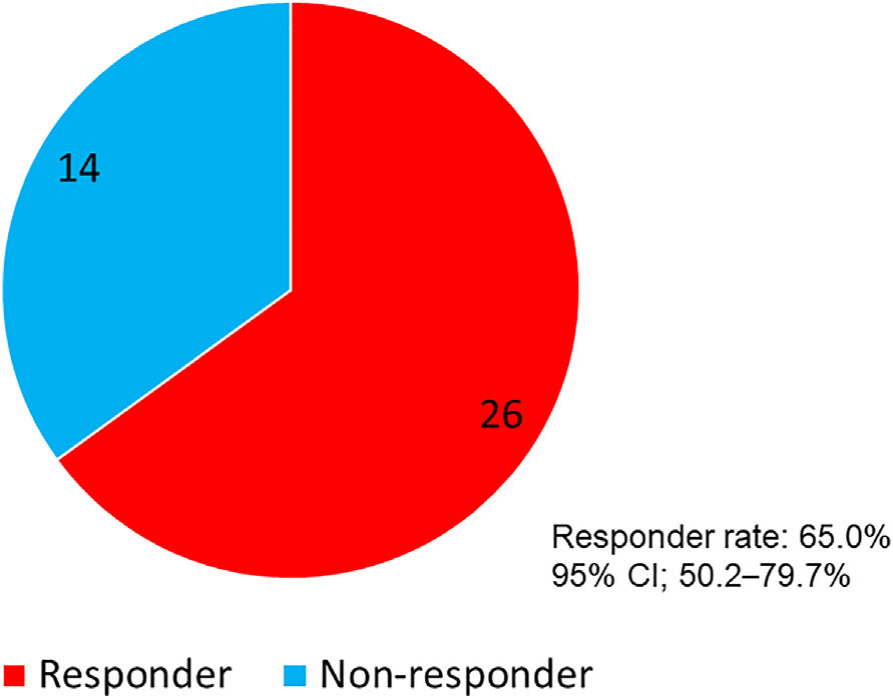
Pie chart showing responders and nonresponders after naldemedine administration. Responder rate: 65.0%, 95% CI; 50.2%–79.7%.

The frequency of defecation of all 40 patients was observed for at least seven days before and after naldemedine administration. As shown in Figure 1, 26 (65%, 95% CI: 50.2%–79.7%) patients were responders, and 14 were nonresponders. Table 3 presents the patient characteristics according to the response. There was no statistical difference in patient background between the responders and nonresponders.

**TABLE 3:** Patient characteristics according to response

	Responder (*n* = 26)	Nonresponder (*n* = 14)	Odds ratio	95% CI	*p*‐value
Sex					
Male/female	19/7	11/3	0.74	0.15–3.46	>0.99
Age (years)					
<75 / ≥75	16/10	11/3	0.43	0.09–1.95	0.31
PS					
0–2 / ≥3	10/16	6/8	0.83	0.22–3.12	>0.99
Regular dose of opioids (mg/day, morphine equivalent)					
<30 / ≥30	9/17	5/9	0.95	0.24–3.71	>0.99
History of chemotherapy within 21 days prior to naldemedine administration^a^					
Yes/No	7/19	4/10	0.92	0.21–3.91	>0.99
History of abdominal surgery before starting naldemedine					
Yes/No	3/23	5/9	0.23	0.04–1.19	0.10
History of radiation to the abdomen and pelvic region before starting naldemedine					
Yes/No	7/19	0/14	NA	NA	0.07
Presence of diabetes mellitus					
Yes/No	6/20	0/14	NA	NA	0.07
Use of laxatives before starting naldemedine administration					
Yes/No	23/3	13/1	0.58	0.05–6.26	>0.99
Use of laxatives after starting naldemedine administration					
Yes/No	23/3	13/1	0.58	0.05–6.26	>0.99

Abbreviations: CI, confidence interval; PS, performance status; NA, not applicable.

*Note*: Cisplatin + vinorelbine (cytotoxic drug) 6 days before the start of naldemedine.

*Note*: Carboplatin+paclitaxel (cytotoxic drug) 7 days before the start of naldemedine.

*Note*: Docetaxel+ramucirumabl (cytotoxic drug) 5 days before the start of naldemedine.

*Note*: S‐1 (cytotoxic drug) 7 days before the start of naldemedine.

*Note*: Gefitinib (tyrosine kinase inhibitor) 5 days after starting naldemedine.

*Note*: Nivolumab (immune checkpoint inhibitor) 1 day before the start of naldemedine pembolizumab (immune checkpoint inhibitor) 3 days before the start of naldemedine.

^a^ Information on whether drug therapy was administered 7 days before and after nardemedine administration and the details of that chemotherapy are listed below as follows.

**FIGURE 2:**
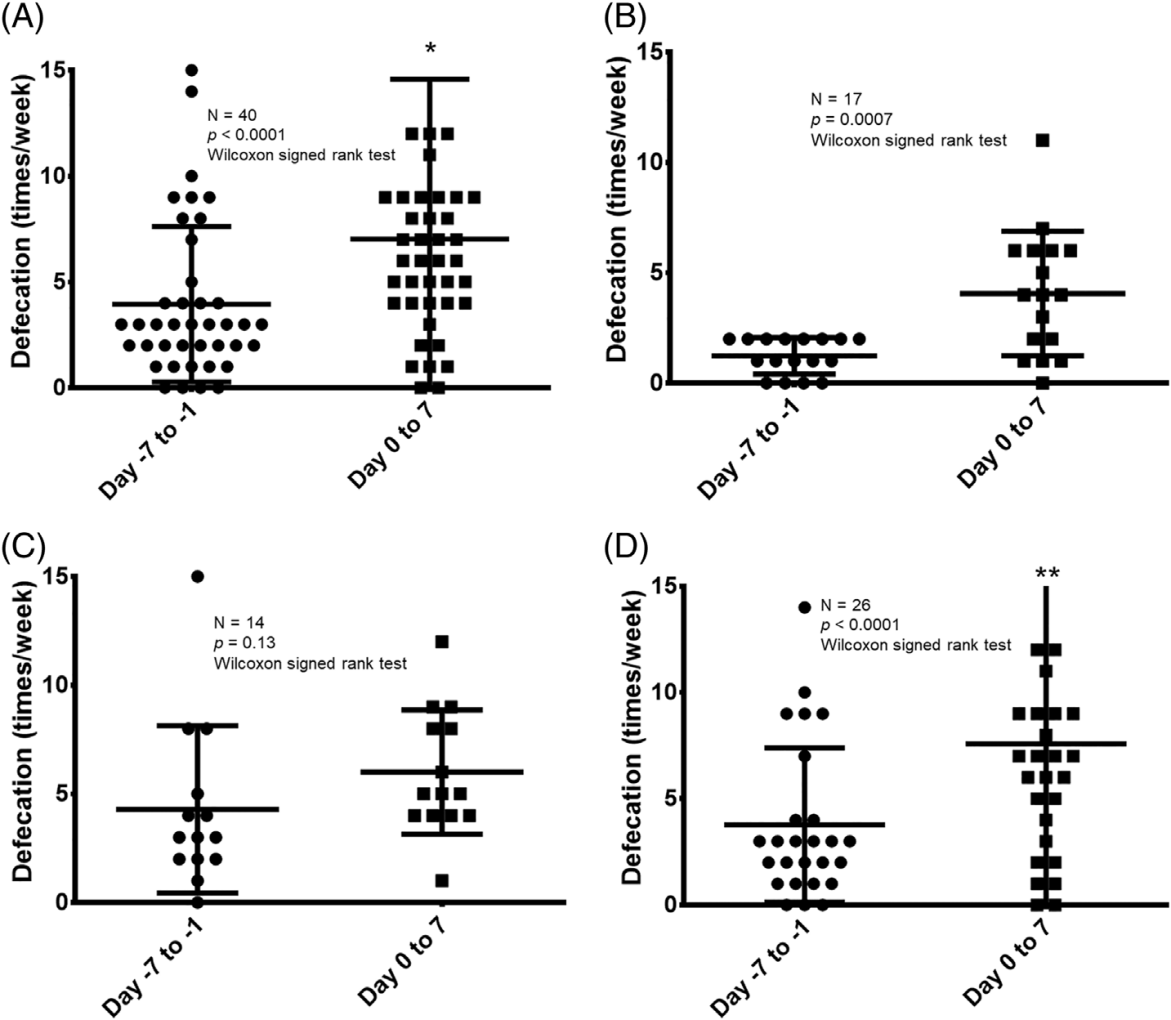
Comparison of defecation frequency seven days before and after naldemedine administration. (a) Comparison of the frequency of defecation before and after naldemedine administration in all patients (*n* = 40). *One patient data point is outside the axis limits. (b) Comparison of defecation frequency before and after naldemedine administration, limited to patients with defecation frequency less than 3 times/week before naldemedine administration (*n* = 17). (c) Comparison of defecation frequency before and after naldemedine administration, limited to patients with less than 30 mg/day of morphine equivalent (*n* = 14). (d) Comparison of defecation frequency before and after naldemedine administration, limited to patients with more than 30 mg/day of morphine equivalent (*n* = 26). **one patient data point is outside the axis limits

Next, we evaluated the change in the frequency of defecation before and after naldemedine administration in the following groups: all patients, only those who defecated less than three times in the week before naldemedine administration, those who were administered <30 mg/day of morphine equivalent, and those who were administered ≥30 mg/day of morphine equivalent (Figure 2). First, in the overall population (*n* = 40), the median number of defecations in the seven days before and after naldemedine administration was 3 (range: 0–14) and 6 (range: 0–49), respectively; thus, the number of defecations increased significantly after naldemedine administration (*p* < 0.0001; Figure 2a). Then, we compared the frequency of defecation during the seven days before and after naldemedine administration in patients who had fewer than three defecations in the week before naldemedine administration (*n* = 17). The median number of defecations during the seven days before and after naldemedine administration was 1 (range: 0–2) and 4 (range: 0–11), respectively; thus, the number of defecations increased significantly after naldemedine administration (*p* = 0.0007; Figure 2b). Next, we compared the frequency of defecation according to the opioid dose. In patients that received <30 mg/day of morphine equivalent (*n* = 14), the median number of defecations in the seven days before and after naldemedine administration was 3 (range: 0–15) and 5 (range: 1–12), respectively; thus, the number of defecations did not increase significantly after naldemedine administration (*p* = 0.13; Figure 2c). Finally, the evaluation was limited to only patients that received ≥30 mg/day of morphine equivalent (*n* = 26). The median number of defecations in the seven days before and after naldemedine administration was 3 (range: 0–14) and 6.5 (range: 0–49), respectively; thus, the number of defecations increased significantly after naldemedine administration (*p* < 0.0001; Figure 2d).

The adverse events judged to be causally related to naldemedine administration are shown in Table 4. Diarrhea was the most common adverse event of any grade, occurring in 11 patients (27.5%), of which nine (81.8%) were grade 1 or 2. No patient experienced grade 4 or higher adverse events.

**TABLE 4:** Adverse events during naldemedine administration

Adverse events^a^	Grade 1	Grade 2	Grade 3	Grade 4
Diarrhea	8	1	2	0
Abdominal pain	0	0	0	‐
Nausea	5	0	0	‐
Vomiting	1	1	0	0
Anorexia	8	0	0	0
Fatigue	5	0	0	‐

^a^ Adverse events were graded using the Common Terminology Criteria for Adverse Events version 5.0.

### Clinical factors influencing treatment response

Next, we analyzed the relationship between the efficacy of naldemedine and various clinical factors using multivariate logistic regression analysis (Table 5). We performed a multivariate logistic analysis utilizing factors of clinical interest: age, PS, morphine equivalent regular opioid dose, history of chemotherapy within 21 days prior to naldemedine administration. Age, PS, morphine equivalent regular opioid dose, and history of chemotherapy within 21 days prior to naldemedine administration did not demonstrate a statistically significant difference in naldemedine efficacy.

**TABLE 5:** Multivariate logistic regression analysis for factors indicative of response in patients receiving naldemedine

Variables	Odds ratio	95% CI	*p*‐value
Age (years)			
<75 / ≥75	2.33	0.52–13.06	0.27
PS			
0–2 / ≥3	1.00	0.24–4.04	0.99
Regular dose of opioids (mg/day, morphine equivalent)			
<30 / ≥30	1.10	0.26–4.52	0.88
History of chemotherapy within 21 days prior to naldemedine administration			
Yes/No	0.91	0.18–4.08	0.60

Abbreviations: CI, confidence interval; PS, performance status.

## DISCUSSION

In this study, we evaluated the frequency of defecation and adverse events in thoracic cancer patients receiving opioids who were hospitalized for at least seven days before and after the start of naldemedine. In addition, we evaluated the effects of naldemedine and factors associated with these effects by assessing the change in the number of bowel movements before and after naldemedine initiation.

In the current analysis, 65.0% of the patients were responders, which is comparable to the responder rate in the COMPOSE‐4 trial (71%) and that of a study on naloxegol (73%), a drug with the same peripherally acting μ‐opioid receptor antagonist as naldemedine.^21^, ^25^ Furthermore, in the overall study population, those who defecated less than three times in the week before naldemedine treatment and those administered opioids with a morphine equivalent of ≥30 mg/day had statistically significant increases in defecation frequency after naldemedine treatment. Notably, a statistically significant improvement in the frequency of bowel movements was observed even in the group of patients judged to be constipated with fewer than three bowel movements in the week before naldemedine administration. Thus, this study confirmed that naldemedine might be effective even in thoracic cancer patients with OIC. Naldemedine is generally effective in the initial stage of opioid administration owing to its pharmacological properties, as a low morphine equivalent dose is often administered in the initial stage of opioid treatment. However, there was no statistically significant increase in the frequency of bowel movements in patients that received <30 mg/day of morphine equivalent, which could be attributed to the small patient population (*n* = 13). It is necessary to study this phenomenon in larger sample sizes in the future. In the multivariate logistic regression analysis, none of the factors we evaluated (age, PS, morphine‐equivalent opioid regular dose, or history of chemotherapy within 21 days before naldemedine administration) were found to impact the efficacy of naldemedine. These findings are consistent with previous reports, which identified no baseline patient characteristic that affect naldemedine efficacy in patients with OIC.^26^, ^27^

In the current analysis, 60.0% of patients had a PS ≥3, while the COMPOSE‐4 and COMPOSE‐5 randomized phase III trials of naldemedine in cancer patients presenting with OIC excluded those with a PS ≥3.^21^ Thus, the efficacy and safety of naldemedine in most patients receiving the drug in clinical practice have not been evaluated in prospective clinical trials. Furthermore, although naldemedine is clinically administered to many outpatients, the defecation of such patients is impossible to calculate accurately; thus, the data were limited to inpatients. Inpatient data were more reliable as they were evaluated by healthcare providers, such as physicians, nurses, and pharmacists. Specifically, hospitalization for at least seven days before and after starting naldemedine was necessary to collect and evaluate sufficient data. In addition, because the patients included in this study were hospitalized for complications such as a poor PS or need for concomitant treatments, these findings should be interpreted with caution. This study had a large number of patients with poor PS (2, 3, 4) and shorter survival. Differences in patient backgrounds between current analysis and that of the COMPOSE‐4 and COMPOSE‐5 trials should be taken up in future studies.

Lung cancer has been reported to have a low rate of metastasis to the gastrointestinal tract at 2–5%.^28^, ^29^ Trials involving various malignancies such as gastrointestinal cancers were similar to phase III prospective trials of naldemedine in terms of efficacy and adverse events. However, they carry a patient selection bias in that patients were enrolled if they had no gastrointestinal obstruction and could take medication orally. In terms of age, it has been reported that approximately 50% of patients with lung cancer are 70 years or older.^30^ Older adult patients generally have more complications and lower organ function than younger patients; therefore, treatment‐related toxicities among older adult patients are a notable concern. Although older adult patients are generally excluded from prospective clinical trials, COMPOSE‐4 and ‐5 included patients 20 years of age and older, and no upper age limit was specified.^21^ Additionally, a subgroup analysis in a phase III trial reported that naldemedine was generally effective and well‐tolerated in patients ≥65 years with chronic noncancer‐related pain.^31^ Consistent with this report, our study found no significant difference in naldemedine efficacy between patients older than 75 and those younger than 75, suggesting that naldemedine can be used effectively to treat older adults. In summary, patients with thoracic cancer do not require special considerations regarding the efficacy or adverse events of OIC treatment.

Regarding safety, diarrhea and abdominal pain were the commonly observed adverse events of naldemedine with incidence rates of 19.6%–39.7% and 1.7%, respectively, in prospective clinical trials of cancer patients with OIC.^21^, ^32^ In our cohort, the incidence rate of diarrhea and abdominal pain was 27.5% and 0%, respectively, which was similar to that of the prospective phase III trials. Although our cohort included patients with a PS ≥3, as well as older adults (≥75 years), serious adverse events, including treatment‐related death, were only observed in two cases (5.0%, grade 3 diarrhea), indicating that naldemedine can be safely administered to patients with thoracic cancer in clinical practice.

This study has several limitations. First, the cohort size was small; however, the number of patients was significant considering the specific inclusion criteria of patients with thoracic cancer who were hospitalized and whose defecation frequency was closely monitored by medical professionals for at least seven days before and after starting naldemedine. Second, due to the retrospective nature of the study, objective assessments, such as the Bristol stool form scale,^33^ bowel function index,^34^ and defecation diary, were not available. This limitation could reduce the validity of the data. Third, the decision to begin or discontinue naldemedine treatment was left to the discretion of each physician, allowing for differences due to subjectivity. Finally, the retrospective nature of the study made standardizing the effects of other treatments and concomitant medications impossible.

In conclusion, this study showed that the efficacy and safety of naldemedine for thoracic cancer patients in clinical practice—where the drug is often administered to older adult patients and those with a poor PS—were comparable with those of prospective studies. Thus, naldemedine may be effective and feasible for most thoracic cancer patients.

## CONFLICT OF INTEREST

The authors have no competing interests to declare that are relevant to the content of this article.

## Supporting information


Figure S1
