# Supplementary figures and images for: A retrospective study of the efficacy and safety of naldemedine for opioid‐induced constipation in thoracic cancer patients
Source: Thorac Cancer. 2022 Jul 5;13(16):2301–8. doi: 10.1111/1759-7714.14557 (PMC9376157; doi:10.1111/1759-7714.14557)

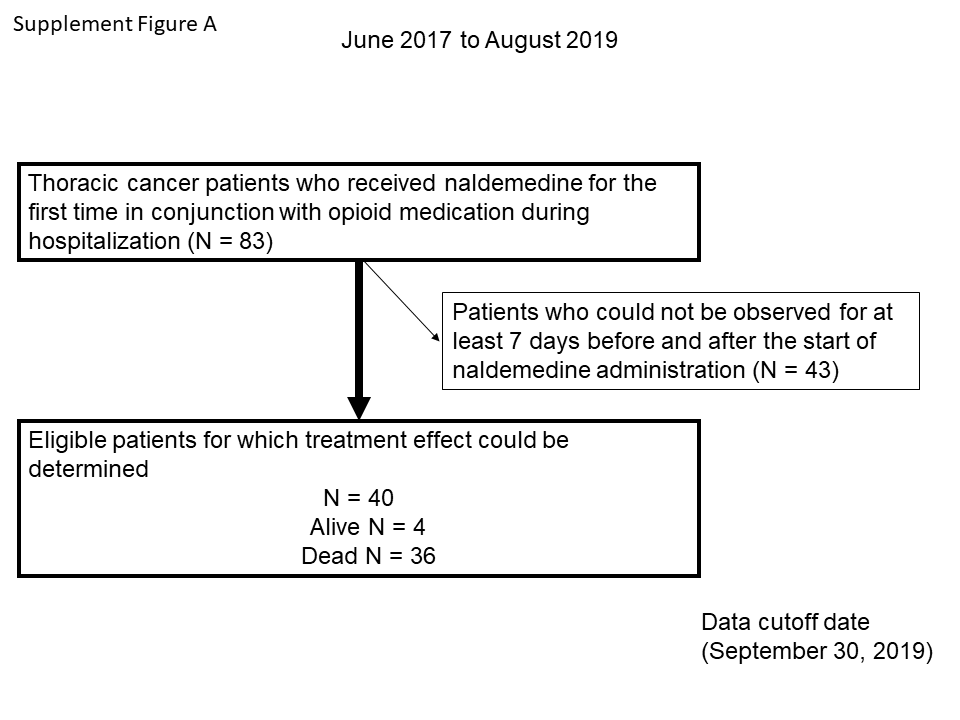

Supplement: Supplementary file 1 — Figure S1 [file TCA-13-2301-s001.TIF]
